# A Smartphone Application Based on Dialectical Behavior Therapy Skills for Binge Eating Episodes: Study Protocol for a Randomized Controlled Trial

**DOI:** 10.3390/healthcare13141749

**Published:** 2025-07-19

**Authors:** Telma Cruz, Tiago B. Ferreira, Debra L. Safer, Cristiana Duarte, Mariana V. Martins

**Affiliations:** 1 Center for Psychology, University of Porto, 4200-135 Porto, Portugal; 2 Faculty of Psychology and Education Sciences, University of Porto, 4200-135 Porto, Portugal; 3 Department of Social and Behavioral Sciences, University of Maia, 4475-690 Maia, Portugal; 4 Department of Psychiatry & Behavioral Sciences, Stanford University School of Medicine, Stanford, CA 94305, USA; 5 School of Education, Language and Psychology, York St John University, York YO31 7EX, UK

**Keywords:** binge eating, smartphone app, dialectical behavior therapy, app intervention, randomized controlled trial, protocol

## Abstract

**Background/Objectives**: With the rapid progression of technology, applications have been proposed as a promising alternative to conventional psychotherapeutic treatment. Nonetheless, research on unguided self-help applications for binge eating remains scarce, with most existing studies utilizing cognitive behavioral therapy (CBT) principles. Therefore, this paper presents the protocol for a randomized controlled trial designed to evaluate the efficacy and acceptability of eMOTE, a standalone application designed specifically for women in Portugal who binge eat. eMOTE, adapted from dialectical behavior therapy (DBT), is unique in that it focuses on teaching emotion regulation skills while also integrating core CBT strategies. **Methods**: At least 68 females who self-report binge eating episodes will be randomized into an intervention group with access to eMOTE for eight weeks or a delayed waitlist, which will have access to eMOTE after the T1 assessment. Assessments will be conducted at baseline (T0), post-intervention (T1), and at 2-month follow-up (T2). The primary outcomes will include objective and subjective binge eating frequency and binge eating symptomatology, while secondary outcomes will assess global levels of ED psychopathology, shape concern, weight concern, eating concern, dietary restraint, compensatory behaviors, mindfulness, emotion regulation difficulties, intuitive eating, psychological distress, and body mass index. **Conclusions**: This study will contribute to the limited literature on the use of smartphone technology as an alternative to traditional psychotherapy. Furthermore, this standalone application will offer insights into the use of emotion regulation and food monitoring components designed for adult females experiencing binge eating episodes.

## 1. Introduction

Objective binge eating is a core diagnostic symptom of bulimia nervosa (BN) and binge eating disorder (BED). It is defined by a sense of loss of control over eating during a specific timeframe, accompanied by the consumption of an unusually large amount of food compared to what most persons would eat in similar circumstances, and is frequently associated with feelings of shame, guilt, and depression [1,2]. BN is estimated to affect 0.1% to 2% of the population, while the lifetime prevalence of BED is approximately 0.2% to 4.7% [3], with both conditions being more prevalent in females than in males [1,4,5]. However, subthreshold presentations of eating disorders (EDs) have been found to be even more prevalent, with individuals reporting levels of ED psychopathology similar to those with full-threshold disorders [6,7,8], highlighting the need for appropriate clinical attention.

Cognitive behavioral therapy (CBT) is a widely recognized and well-validated treatment for EDs [9,10]. CBT-E is transdiagnostic in nature, suggesting that distinct EDs share common underlying mechanisms [2]. While effective, cessation rates for binge eating episodes and compensatory behaviors following CBT-E are only around 50% at post-treatment [10], highlighting the importance of exploring alternative treatment models that address negative affect and emotion dysregulation, as these factors are involved in the development and maintenance of binge eating [11,12,13].

One specialized treatment for emotion dysregulation is dialectical behavior therapy (DBT), initially developed for patients diagnosed with borderline personality disorder and suicidal behaviors or a pattern of ongoing self-harm [14]. Adaptations of the original version have been made for EDs [15,16,17], maintaining the core skills of mindfulness, emotion regulation, and distress tolerance, and also emphasizing difficulties in regulating emotions as a key mechanism. According to the affect dysregulation model, individuals engage in binge eating to cope with intense emotions that they are unable to regulate through other means. A growing body of evidence supports the efficacy of DBT in treating binge eating by improving emotion regulation, increasing treatment retention, and achieving abstinence rates ranging from 64% to 89% [18,19]. Self-help adaptations have also shown promising results [20,21].

Mindfulness, a key component of DBT, may also support more adaptive eating behaviors, such as intuitive eating, which involves being attuned to internal hunger and satiety signals and eating according to these cues [22,23]. There is evidence that individuals with higher levels of intuitive eating tend to show fewer binge eating symptoms and other disordered eating behaviors [24,25].

Nonetheless, because DBT and CBT require extensive training and multiple sessions, treatment can be difficult due to high costs and limited availability. In addition, research has shown that many individuals with EDs do not receive proper care, with only 32% seeking treatment from a health professional [26]. Smartphone applications (apps) have been proposed as a way to address this gap by providing evidence-based principles in a less intensive, cost-effective, and more accessible format for those who cannot receive traditional treatment [27].

Previous randomized controlled trials (RCTs) have demonstrated that app interventions can be efficacious for EDs, leading to significant reductions in binge eating episodes and overall ED psychopathology, both in guided self-help formats [28] and when used as standalone interventions [29]. While most apps have been based on CBT principles, a recent RCT [30] evaluated the efficacy of a DBT-based app and reported significant reductions in objective binge eating and other key ED symptoms, along with improvements in mindfulness. Considering these findings, an app incorporating emotion regulation components also seems to be a promising tool for managing binge eating and related psychopathology, as an alternative to those developed based on CBT principles.

We developed eMOTE, a DBT skills-based app that also incorporates some key CBT components, designed to address objective and subjective binge eating in women. We also included subjective binge eating, defined as a sense of loss of control over eating without consuming an objectively large amount of food, as growing evidence suggests that it is associated with levels of ED psychopathology comparable to those observed in objective binge eating [31,32]. Unlike the app developed by Linardon et al. [30], which focuses exclusively on DBT, eMOTE combines DBT skills with specific CBT components, including dietary restraint and self-monitoring. Furthermore, we focused on females, not only due to the higher prevalence of binge eating in women [3,4,5], but also due to the greater likelihood for women to seek treatment [33], allowing the app to be more specifically tailored.

### Objectives and Hypotheses

The main objectives of this RCT are to evaluate whether this digital intervention demonstrates superiority over a delayed waitlist (DWL) condition in reducing binge eating and key ED symptoms, emotion regulation difficulties, and psychological distress, as well as in improving mindfulness and intuitive eating, and to assess its acceptability. Participants will be randomly allocated to an intervention group (IG) or a DWL group, with the trial taking place in Portugal. Considering the objectives, we hypothesize the following:

1. At the end of the intervention (T1), the IG will show improvements in the primary outcomes: objective and subjective binge eating frequency and binge eating symptomatology, compared to the DWL;
2. At the end of the intervention (T1), the IG will show improvements in the secondary outcomes, including global levels of ED psychopathology, shape concern, weight concern, eating concern, dietary restraint, compensatory behaviors, mindfulness, emotion regulation difficulties, intuitive eating, and psychological distress, compared to the DWL. No significant differences in body mass index (BMI) are expected between groups;
3. Improvements observed in the IG at T1 will be maintained at follow-up (T2);
4. The DWL group will also show improvements in primary and secondary outcomes after having access to the 8-week intervention (T2);
5. Users will rate the app as acceptable.

## 2. Materials and Methods

The present protocol followed the Standard Protocol Items: Recommendations for Interventional Trials (SPIRIT) guidelines [34], and the RCT is registered on ClinicalTrials.gov (Identifier: NCT06683456).

### 2.1. Trial Design

A prospective RCT with two parallel arms will be conducted to evaluate the efficacy of a standalone DBT skills-based app for binge eating, using a 1:1 allocation ratio. Participants meeting the eligibility criteria will be randomly assigned to either the IG or the DWL group. The use of a DWL control group aligns with methodologies adopted in previous trials of standalone app-based interventions targeting key ED symptoms [29,30]. Data will be collected at baseline (T0), at the end of the 8-week intervention (T1), and at 2-month follow-up (T2). The DWL group will be evaluated at baseline (T0) and will remain on a waitlist until the T1 assessment has been completed. After that, participants in the DWL group will be granted access to the app.

### 2.2. Participant Eligibility Criteria

Participants are eligible if they fulfil the following criteria: (i) female, (ii) aged between 18 and 60 years, (iii) self-report at least one objective or subjective binge eating episode in the past four weeks, measured using the Eating Disorder Examination Questionnaire (EDE-Q) [35], (iv) own a smartphone, and (v) are fluent in Portuguese.

The exclusion criteria include the following: (i) currently attending psychotherapy, (ii) participation in another clinical trial, (iii) a BMI below 18.5, (iv) self-reporting an average of eight or more episodes of inappropriate compensatory behaviors and/or objective binge eating per week, assessed by the EDE-Q [35], (v) being pregnant, or (vi) self-reporting a diagnosis of bipolar disorder, psychotic disorder, active suicidal ideation, substance abuse, or borderline personality disorder.

### 2.3. Recruitment

This nationwide study conducted in Portugal will use a recruitment strategy involving social media, national press, and the University of Porto (institutional emails and posters), with the aim of maximizing reach. On the project’s social media, the focus will be on reaching adult women, using headlines such as “Do you find it difficult to control your eating?”, “Do you feel like you eat compulsively?”, and “Do you rely on food to cope with distress?”. These posts will also provide information about the app as a digital intervention and the target audience. Additional details about the study will be shared through other types of content.

Women interested in participating will be directed to a registration link on the LimeSurvey platform, where detailed information will be presented about the study’s objectives, data protection, and information about the research team. Those who agree to participate in the study will provide their informed consent online by selecting an option indicating their understanding of and agreement with all study procedures and their voluntary participation. Participants will then complete the sociodemographic and clinical questionnaire and the baseline assessment (T0) provided online through the same platform. Eligibility will be assessed by a research team member.

Eligible participants will receive an email with further instructions, inviting them to attend an online orientation meeting via the Zoom platform. Individuals who do not meet the eligibility criteria will be emailed an explanation of the study’s inclusion and exclusion criteria, and recommendations for seeking professional support will be specified.

### 2.4. Randomization Procedure

The randomization process will follow a 1:1 allocation ratio and will be conducted by an independent researcher using Matlab 2024a software to generate a randomization protocol. This approach ensures the integrity of sequence generation and allocation concealment while keeping the researchers involved in the trial blinded to the randomization process.

### 2.5. Study Conditions

An online orientation meeting will be conducted via the Zoom platform to review the study’s objectives, and participants will be informed as to whether they will have access to the app (IG) or be placed on a DWL, depending on their group allocation.

At this time, participants in the IG will be guided on downloading the app to their smartphones via a provided link. An account will be created for each participant using their personal identification number, ensuring that the app functions correctly. A brief video tutorial demonstrating app navigation will then be presented, and guidelines for its use will be given. The IG will have access to eMOTE for eight weeks and will receive two follow-up emails during the intervention, approximately one week and one month after starting, to check whether they are experiencing any technical difficulties using the app and to offer procedural support if needed. After this period, participants will no longer have access to their accounts and will receive an email inviting them to complete the post-intervention assessment (T1). The follow-up assessment (T2) will be sent two months later.

Participants in the DWL group will be informed during the initial online meeting that they will have the opportunity to access eMOTE after completing the T1 evaluation. Afterwards, the same procedures followed for the IG will be applied.

**Figure 1:**
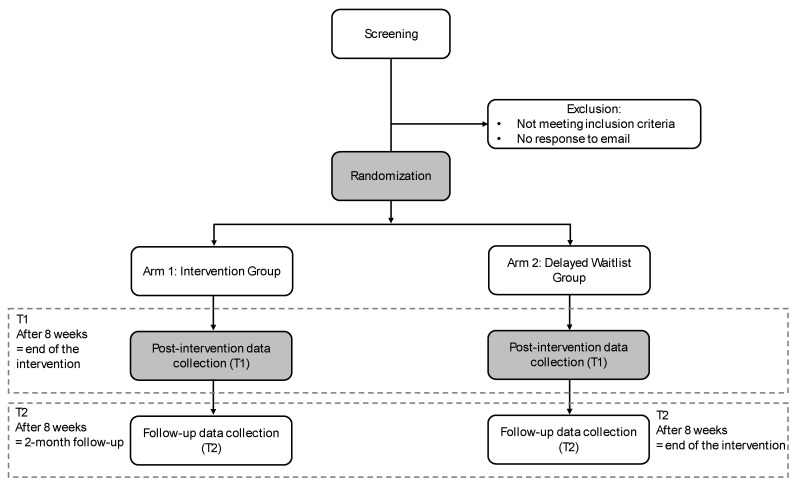
Diagram of participant enrolment.

After all assessments have been completed, participants will receive an email expressing gratitude for their contribution to the study. In the case of participants informing the research team that they have initiated psychotherapy, no further data will be considered from that point onward. The flow of participants through the trial stages is illustrated in Figure 1.

### 2.6. eMOTE Intervention

The eMOTE app is a digital intervention in Portuguese for binge eating, delivered through a mobile device and compatible with both iOS and Android systems. The intervention is grounded in an empirically supported treatment, DBT skills, that were adapted from a manual intended for professionals on BN and BED [15], and from a self-help book addressing binge eating and out-of-control eating [36]. The primary aim of eMOTE is to teach and encourage the regular practice of specific skills and strategies to cope with emotional difficulties without resorting to destructive behaviors, such as binge eating and/or purging.

eMOTE includes four modules: psychoeducation, mindfulness skills, emotion regulation skills, and distress tolerance skills, as well as two self-monitoring diaries: the food diary and the emotion diary.

The psychoeducation module comprises content derived from CBT [37] and DBT [36], delivered through brief videos featuring psychologists and text. Its goal is to increase the understanding of binge eating, the mechanisms that perpetuate it, and the role of emotion regulation. Topics covered in this module include binge eating episodes, compensatory behaviors, the role of dietary restraint, emotion regulation, the biosocial theory, and the utility of emotion and food diaries. The second module integrates mindfulness and emphasizes observing experiences without judgment and learning to describe emotional states, thoughts, and urges as they arise in the present moment [15,36]. The third module focuses on providing emotion regulation skills to decrease vulnerability to intense emotions, increase positive emotions, practice the radical acceptance of emotions, and shift emotional states [15,36]. Finally, the fourth module, distress tolerance skills, aims to deliver strategies to cope with pain and distress in the present moment (that cannot be immediately alleviated) without engaging in binge eating behaviors, and promoting acceptance and skillful responses to prevent worsening emotions [15,36]. As part of the intervention, a final short video was incorporated to congratulate participants on completing the intervention and acknowledge their effort, while emphasizing the importance of regularly practicing DBT skills and reinforcing the commitment to stop binge eating. The skills content is delivered through text, audio recordings, and videos.

The food diary was developed by integrating principles from CBT [37] and DBT [15], allowing participants to track their eating patterns, including ED behaviors, record whether mindful eating was practiced, and any additional comments. The emotion diary was adapted based on the diary cards [15] and offers a space to record feelings, identify which skills were most helpful for managing those feelings, and reflect on the day and the exercises completed. Additionally, content related to compensatory behaviors will only be available to participants who have reported these symptoms.

The app’s four modules follow a sequential structure, unlocking progressively only after completing the previous one. Furthermore, progress can be monitored by reviewing binge episodes over the past seven days, the associated emotions, and module progression. A favorites page is also available for easy access to bookmarked exercises. Two default prompts are set to boost engagement with the app, which can be customized to receive up to six daily reminders. The app collects active data entered by users, as well as engagement metrics, including login activity, time spent on modules and exercises/skills, and the number of diary entries. Screenshots of eMOTE are available in the Supplementary Materials.

Although eMOTE is a standalone treatment, meaning it is self-guided, participants will be encouraged to use the app every day and practice the DBT skills daily.

### 2.7. Development and Adaptation of the Intervention

Several aspects were carefully considered during the development of the digital intervention. First, psychoeducation and DBT skills were adapted for delivery through a mobile device by a researcher with experience in CBT models and then translated into Portuguese. Afterwards, the content was reviewed by two international experts and two females with binge eating behaviors. A co-creation approach was adopted for developing the app prototype, involving meetings with a psychologist, a designer, two women with lived experience of binge eating, and an engineer. Lastly, the app was evaluated by experts and users with binge eating episodes. This process led to the refinement of the prototype, resulting in the final version of eMOTE.

### 2.8. Measures

The schedule of enrollment, interventions, and assessments is presented in Table 1.

#### 2.8.1. Sociodemographic and Clinical Data

The sociodemographic and clinical questionnaire will assess age, level of education, marital status, duration of binge eating, previous treatment for ED symptomatology, and prior psychiatric diagnoses.

#### 2.8.2. Primary Outcomes

Objective and subjective binge eating frequency will be measured using the 36-item Eating Disorder Examination Questionnaire (EDE-Q) [35,38]. This self-report tool evaluates the frequency of binge eating over the past 28 days, along with other key aspects of ED psychopathology.

Binge eating symptomatology will be evaluated with the Binge Eating Scale (BES) [39,40], a 16-item self-report questionnaire that assesses binge eating symptoms, covering emotional, cognitive, and behavioral aspects. Each item presents three to four statements, and participants are asked to select the one that best represents their experience. Responses are rated on a scale from 0 (indicating no difficulties with binge eating) to 3 (severe difficulties with binge eating), with higher scores indicating more severe levels of binge eating.

#### 2.8.3. Secondary Outcomes

Global levels of ED psychopathology, shape concern, weight concern, eating concern, dietary restraint, and compensatory behaviors will be assessed using the 36-item EDE-Q [35,38]. The EDE-Q is a widely used measure for evaluating the frequency of core behaviors and attitudinal features associated with ED pathology over the past 28 days. Respondents rate each item on a 7-point scale varying from 0 (not at all or no days) to 6 (markedly or every day), and ED behaviors are measured based on their presence and frequency, with higher scores reflecting greater levels of disordered eating.

Mindfulness will be measured using the Mindful Attention and Awareness Scale (MAAS) [41,42], a self-report measure that evaluates individual differences in mindfulness as a dispositional trait, focusing on attention and awareness of present-moment experiences in daily life, particularly among individuals without prior meditation experience. The scale comprises 15 items rated on a 6-point scale, ranging from 1 (almost always) to 6 (almost never), and higher scores indicate greater levels of mindfulness.

Emotion regulation difficulties will be assessed using the Difficulties in Emotion Regulation Scale (DERS) [43,44], a 36-item self-report measure designed to assess emotion dysregulation. The scale includes six subscales: (1) nonacceptance of emotional responses, (2) difficulties engaging in goal-directed behavior, (3) impulse control difficulties, (4) lack of emotional awareness, (5) limited access to emotion regulation strategies, and (6) lack of emotional clarity. Items are answered on a 5-point scale varying from 1 (almost never) to 5 (almost always), and higher scores indicate more significant difficulties in emotion regulation.

Intuitive eating will be assessed using the Intuitive Eating Scale-2 (IES-2) [45,46], a 23-item instrument that involves four subscales: (1) unconditional permission to eat, (2) eating for physical rather than emotional reasons, (3) reliance on internal hunger and satiety cues, and (4) body–food choice congruence. Items are rated on a 5-point scale, ranging from 1 (strongly disagree) to 5 (strongly agree), with higher scores representing more adaptive eating behaviors.

Psychological distress will be measured using the Depression Anxiety Stress Scales-21 (DASS-21) [47,48], a well-established 21-item tool that evaluates symptoms of depression, anxiety, and stress over the past week. Participants rate each item on a 4-point scale varying from 0 (did not apply to me at all) to 3 (applied to me very much, or most of the time), with higher scores corresponding to more emotional distress in each domain.

The body mass index will be calculated using the following formula: weight (in kilograms) divided by the square of the height (in meters).

#### 2.8.4. Acceptability

Acceptability will be assessed by evaluating the utility of and satisfaction with eMOTE. At the end of each module, participants will use the app to rate its utility and their satisfaction on a 5-point scale, ranging from 1 (not at all) to 5 (extremely).

#### 2.8.5. Adherence

Adherence to eMOTE will be evaluated using the following measures: the number of modules completed, the number of exercises completed, the number of diaries completed, the number of times accessed, and the total time spent using eMOTE.

#### 2.8.6. User Feedback

An open-ended question will be included in the online questionnaires to explore participants’ experiences with the intervention and the app, including what they found most helpful and suggestions for improvement.

### 2.9. Sample Size Calculation

A priori power analysis was performed using G*Power version 3.1 [49] to verify the sample size. The calculation was based on the results from previous trials that explored the efficacy of standalone apps for binge eating [29,30]. G*Power calculations for repeated measures analysis of variance (ANOVA) with two groups and two time points (T0-T1), with an alpha set at 0.05, a statistical power of 0.95, and an effect size of *f* = 0.25, determined a recommended sample size of 54. Considering a 25% attrition rate identified in a meta-analysis of digital interventions for ED [50], a final sample size of 68 participants is required.

### 2.10. Statistical Analysis Plan

Data analysis will be performed in SPSS (version 29.0) and R. If needed, the forward imputation method will be used to input the missing data in the ForImp R package [51]. An intention-to-treat analysis will be conducted, including all randomized participants in the statistical analysis [52]. Differences between groups at baseline (T0) will be evaluated using *t*-tests for continuous variables and chi-square tests for categorical variables. Treatment effects from baseline to post-intervention on primary and secondary outcomes will be analyzed using mixed ANOVAs (2 × 2), with the time points as the within-group factor and the group as the between-group factor. To assess whether the effects of the intervention will be maintained from post-intervention (T1) to follow-up (T2), repeated measures ANOVAs (T1 vs. T2) will be performed for the IG. In addition, for the DWL condition, repeated measures ANOVAs (T1 vs. T2) will be used to evaluate the changes after receiving access to the app. A reliable and clinically significant change index will be computed to determine the proportion of participants that reliably changed and recovered. Dropout and deterioration rates will be assessed. Number-needed-to-treat will be computed. Where adequate, 95% confidence intervals will be estimated. R packages ggstatsplot [53] and ggplot2 [54] will be used for data analysis and visualization.

### 2.11. Data Management

Participant data will be handled and stored in compliance with applicable laws and regulations, including the EU General Data Protection Regulation (GDPR). A pseudonymization procedure, which separates participants’ identification (names and email addresses) from collected data, will be used. Names and email addresses will only be accessible to a designated member of the research team. To make data anonymous, they will be deleted as soon as they are no longer needed. To pair them with collected data, they will be referenced using a unique six-digit personal identification number chosen by the participants. Data obtained from the online questionnaires and the app will be encrypted and restricted to access by the research team members only. The survey data will be gathered through the LimeSurvey platform provided by the University of Porto, which complies with the requirements of the GDPR. Participants will register and access eMOTE using their personal identification number, guaranteeing that no personal identifiers are stored on app servers. Encrypted data will be used exclusively for scientific research purposes, with all information securely stored on password-protected laptops and databases to safeguard its protection.

### 2.12. Ethics and Dissemination

The study will comply with the ethical principles delineated in the World Medical Association’s Declaration of Helsinki and has received approval from the Ethics Committee of the Faculty of Psychology and Education Sciences at the University of Porto (Ref. 2023/04-01b).

Participants will provide informed consent after being fully informed about the objectives and procedures of the study. In addition, the smartphone app will also present its terms and conditions, which contain a disclaimer specifying who should not use eMOTE. As participants will not have access to other forms of psychological intervention during the study, the app will include a section containing contact details for treatment services, as well as for the research team. Furthermore, individuals who self-report symptoms consistent with severe or extreme cases of BN or BED, as defined by the severity levels in the DSM-5 [1], a BMI below 18.5, or other risks associated with participation, will be excluded, as these cases may require more specialized or intensive care. All excluded participants will receive an email from the research team outlining the criteria for the trial, alongside recommendations to seek professional support.

The findings from this study will be presented at international and national scientific conferences and published in peer-reviewed journals.

## 3. Discussion

This paper outlines the protocol of a RCT designed to evaluate the efficacy and acceptability of the eMOTE app. This is the first study protocol that details the trial procedures of a standalone app that combines DBT skills with selected CBT components. The app is intended to support the development of skills that improve emotion regulation and present-moment awareness, with the aim of promoting healthier eating behaviors and potentially reducing ED psychopathology and psychological distress. As a digital intervention, it offers continuous access to these skills and allows real-time diary entries. Its self-monitoring features are designed to ensure greater privacy and boost user engagement in completing logs [55]. Our trial also aims to address the substantial treatment gap in ED care, as research shows that only a minority of individuals seek support from qualified health professionals [26], and apps offer promising potential to bridge this gap [27,29].

The strengths of our study include the innovative nature of the app, which integrates DBT with specific CBT content (dietary restraint and self-monitoring), given that both DBT and CBT are evidence-based approaches for the treatment of EDs [2,15]. To date, only one recent study has evaluated the efficacy of an app intervention grounded in similar principles for recurrent binge eating [30]. Unlike eMOTE, that intervention was developed exclusively using DBT principles, and was not specifically designed for women. Furthermore, this trial will provide a 2-month follow-up to assess whether the effects of this preliminary study are sustained over time, addressing a gap in the prior study [30], which did not include a follow-up assessment point for participants without access to the app. Lastly, another strength is that the development process of eMOTE was informed by feedback from women with lived experience of binge eating, ensuring that it is user-friendly and adapted to meet the needs of the target population.

This study also presents limitations. A high attrition rate is anticipated, as observed in other app-based interventions for EDs [29,30]. Strategies have been employed to help reduce dropouts, such as app prompts and two follow-up emails that will be sent during the intervention. Although self-report measures may enhance scalability and ecological validity, they also present limitations that may affect the estimation of symptom frequency [56,57] and treatment effects. In addition, the app does not include screenings to detect significant symptom deterioration or automatically alert users, which may limit the ability to monitor symptom worsening in real time. To help mitigate this limitation, participants are informed that they can withdraw from the study at any time and that the app is not a substitute for professional treatment. Referral contacts for mental health services are also available within the app for participants who feel that they need additional support. Also, eMOTE is designed specifically for adult women, which limits the generalizability of the results to other age groups and gender identities. Furthermore, because eMOTE was developed in Portugal and in Portuguese, the protocol cannot be considered a global protocol without additional research attention focused on potential differences between those in Portugal and other settings. Lastly, since blinding is challenging in trials involving apps, participants in the DWL group will be aware that they will have access to the app at a later stage, which may influence their behavior due to allocation knowledge.

## 4. Conclusions

The findings from this RCT will help to assess whether eMOTE is a potentially efficacious and acceptable intervention for managing binge eating and related psychopathology, as well as for promoting adaptive emotion regulation skills. The study also aims to contribute to the limited literature on standalone app interventions for binge eating, with a focus on emotion regulation and self-monitoring features designed for adult women.

## Figures and Tables

**Table 1:** Schedule of enrolment, interventions, and assessments.

	STUDY PERIOD			
	Enrolment	Baseline	Post-allocation	
TIMEPOINT	*T-1*	*T0*	*T1*	*T2*
**ENROLMENT:**				
**Eligibility screen**		X		
**Informed consent**	X			
**Allocation**		X		
**INTERVENTIONS:**				
***Intervention*** ***group***		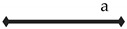		
***Delayed waitlist group***				
**ASSESSMENTS:**				
***Demographic/clinical data***		X		
***EDE-Q***		X	X	X
***BES***		X	X	X
***MAAS***		X	X	X
***DERS***		X	X	X
***BMI***		X	X	X
***IES-2***		X	X	X
***DASS-21***		X	X	X
***Acceptability***		Following each module		
***User feedback***				X

*Note*. ^a^ only for the intervention group. Abbreviations: app, application; EDE-Q, Eating Disorder Examination Questionnaire; BES, Binge Eating Scale; MAAS, Mindful Attention and Awareness Scale; DERS, Difficulties in Emotion Regulation Scale; BMI, body mass index; IES-2, Intuitive Eating Scale-2; and DASS-21, Depression Anxiety Stress Scales-21.

## Data Availability

The data that support the findings of this study are available from the corresponding author, T.C., upon reasonable request after the publication of findings.

## Supplementary Materials

The following supporting information can be downloaded at: https://www.mdpi.com/article/10.3390/healthcare13141749/s1, eMOTE App—Illustrative Screenshots.
