# Supplementary material for: A Smartphone Application Based on Dialectical Behavior Therapy Skills for Binge Eating Episodes: Study Protocol for a Randomized Controlled Trial
Source: Healthcare (Basel). 2025 Jul 19;13(14):1749. doi: 10.3390/healthcare13141749 (PMC12294396; doi:10.3390/healthcare13141749)
Supplement: Supplementary file 1 [file healthcare-13-01749-s001.zip › healthcare-3582124-supplementary.pdf]

Supplementary Materials

<u>eMOTE App – Illustrative Screenshots</u>

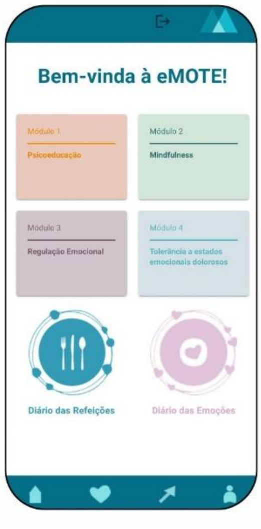

Figure S1. Home page

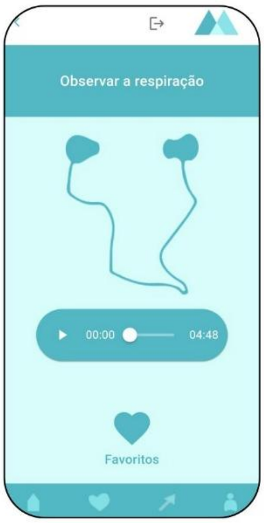

Figure S2. Example of an audio skill

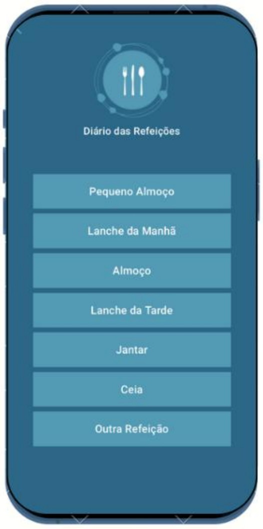

Figure S3. Food diary

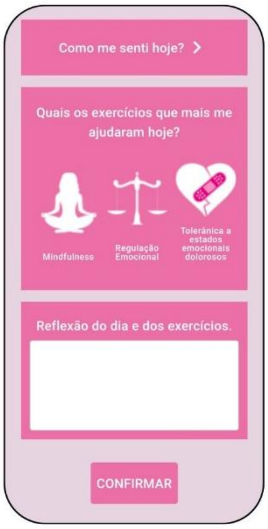

Figure S4. Emotion diary
